# An Assessment of the Accuracy of Recurring Esthetic Dental Proportion: An In Vivo Comparative Analysis of Maxillary Anterior Teeth Proportion With Chu’s Gauge and Custom-Made Calipers

**DOI:** 10.7759/cureus.49713

**Published:** 2023-11-30

**Authors:** Prakyath Malli, Shrimaa B Kateel, Amal K, Sanath Kumar Shetty, Tripthi P Shetty, Uma Mayur Prabhu

**Affiliations:** 1 Department of Prosthodontics, Yenepoya Dental College, Yenepoya (Deemed to be University), Mangalore, IND; 2 Department of Oral and Maxillofacial Surgery, AB Shetty Memorial Institute of Dental Sciences, Mangalore, IND

**Keywords:** teeth proportion, smile engineering, chu's esthetic gauge, smile esthetics, golden proportion

## Abstract

Smile designing, in harmony with nature, has always been a challenge in dentistry. Several theories have been proposed in the past with a view to replicate an esthetic smile. One such method proposed by Dr. Chu involves using the recurring esthetic dental (RED) proportion. He designed a scale for calculating the average values for the height and width of upper anterior teeth for a specified population. However, whether this average is valid for other populations has not been verified. Hence, this study was conducted to evaluate if Chu’s gauge value agrees with the dimension of anterior teeth in the South Indian population. The study involved 362 subjects whose anterior teeth dimension was assessed using Chu’s gauge and a custom-made caliper. The proportion of the population whose dimension aligns with the average values on Chu’s gauge was evaluated. The results showed that in the cohort, 39% had their central incisor dimension coinciding with the red band of Chu’s esthetic scale, 10% had their lateral incisor coinciding with the blue band of Chu’s esthetic scale, and 6.4% of the subjects had their canine dimension coinciding with the yellow band of Chu’s esthetic scale.

## Introduction

A good smile is always an asset as it represents warmth and confidence. In dentistry, patients’ expectations regarding their appearance can often be challenging, and their demands are not always physiologically and mechanically feasible in terms of creating an esthetically captivating smile, which makes smile designing very challenging. A surge in patients' cosmetic needs has led to tremendous advancements in various technologies related to esthetics in dental restoration. Magne and Belser in 1979 proposed a checklist to analyze the fulfilment of any esthetic restoration. This encompassed the incorporation of dental components, gingival esthetics, and greater subjective esthetic integration. The measurement of teeth plays an important role in this scenario [1]. 

For effective smile designing, a thorough knowledge of the facial components, esthetics, proportions, and symmetry of the face is necessary [2]. Lombardi in 1973 proposed that dental and facial esthetics may be optimized if a repeated steady proportion exists in terms of significant incisor-to-lateral-incisor width and lateral incisor-to-canine width. This proportion is termed the golden proportion and amounts approximately to 1.618 to 1 [3]. Based on the golden percentage, the width of the lateral incisor is 62% of the width of the central incisor and the width of the canine from the frontal view is 62% of the lateral. However, this proportion was found only in 17% of the population. Preston proposed that the maxillary lateral incisor width should be 66% of the primary incisor width and canine width must be 84% of the width of the lateral incisors when viewed from the frontal aspect. In 1999, Snow proposed that the width of every maxillary primary incisor should be 25% of the maxillary intercanine distance, lateral incisors need to be 15%, and canines should be 10% to achieve optimal esthetic smile. Lombardi defined continuous percentage or repeated ratio as present among the widths of primary lateral and canine; however, it need not be in 62%. This value is known as recurring esthetic dental (RED) proportion. Studies have shown that this is around 62-80% [3].

Photogrammetric evaluation of RED and golden proportion was performed by Murthy et al., Meshram et al., and Ahmed et al. among the population of central India, and they concluded that there is no evidence of ideal tooth width ratio and data in the literature, which supports their findings overall [4,5,6,7,8,9,10]. Dr. Stephen J Chu [11] devised a device primarily based on the observation of the demographics of the Caucasian population. Chu’s esthetic gauge is based on the idea of recurring esthetic dental (RED) proportion: the use of the 78% RED percentage. As this device was designed primarily based on demographic data, its accuracy for all populations is not always validated.

RED proportion describes a regular ratio between the width and height of maxillary anterior teeth. It states that the widths of successive teeth when regarded from the frontal aspect should continue to be consistent as we move distally [12,13,14]. The purpose of this study was to assess whether the common dimensions of width and height of maxillary anterior teeth in the South Indian population align with the outcomes achieved with the aid of using Chu’s gauge.

## Materials and methods

Methodology

The study was approved by the Scientific Review Board, Yenepoya Dental College (ref no. YDC/SRB/589/F/SS/034/2022). The study included 362 subjects (level of significance: 5%, absolute precision: 5%) reporting to the outpatient section, Department of Prosthodontics, Yenepoya Dental College. The subjects were selected based on specific inclusion and exclusion criteria. Individuals in the age group of 16-32 years with healthy teeth were included. Patients with fractured teeth, those with any history of restoration, those under orthodontic treatment, and those with malaligned teeth, any systemic illness, periodontally compromised teeth, presence of any peg laterals or congenitally missing teeth, presence of diastema, and presence of trauma from occlusion were excluded. Subjects were informed about the procedure and informed consent was obtained.

Assessment using the Chu’s gauge

**Figure 1:**
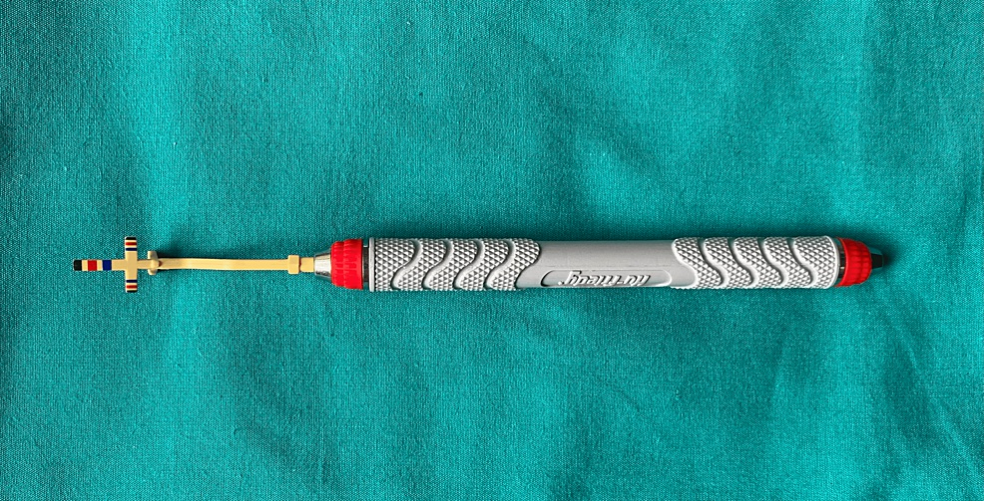
Description of the Chu’s gauge The Chu’s gauge has a T-bar tip (for normally aligned teeth) and an in-line tip (for crowded teeth). The T-bar tip has a horizontal arm, vertical arm, and incisal guide. The measurements on the horizontal arm range from 5.5 mm to 10.5 mm, and on the vertical arm, they range from 7 mm to 13.5 mm, which corresponds to the minimum and maximum width and height of the maxillary anterior. The average width and length for lateral incisors correspond to the blue band (6.5 mm/8.5 mm), that of the canine to the intermediate yellow band (7.5 mm/9.5 mm), and that of the central to the red band (8.5 mm/11 mm)

The Chu's gauge and custom-made caliper were disinfected. The Chu’s gauge consists of two tips (Figure 1). The T-bar tip is for normally aligned teeth. The T-bar tip has a horizontal arm, vertical arm, and incisal guide. Both arms are color-coded. The measurements on the horizontal arm range from 5.5 mm to 10.5 mm. On the vertical arm, they range from 7 mm to 13.5 mm, which corresponds to the minimum and maximum width and height of the maxillary anterior. The average width and length for lateral incisors correspond to the blue band (6.5 mm/8.5 mm), that of the canine to the intermediate yellow band (7.5 mm/9.5 mm), and that of the central to the red band (8.5 mm/11 mm). This instrument is based on 78% RED proportion.

**Figure 2:**
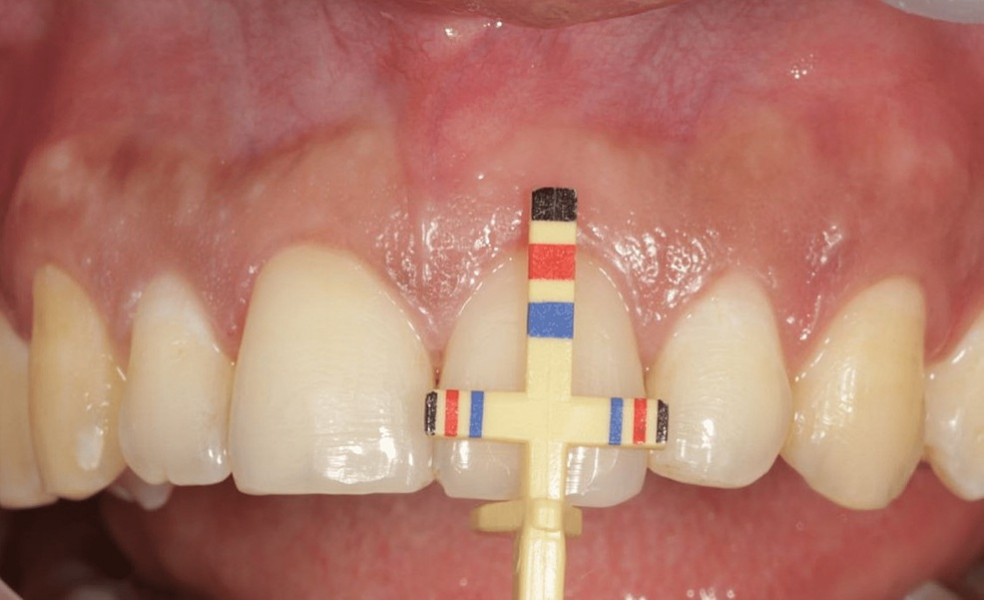
The variation between the color coding on the Chu’s gauge and the dimension of central incisors

**Figure 3:**
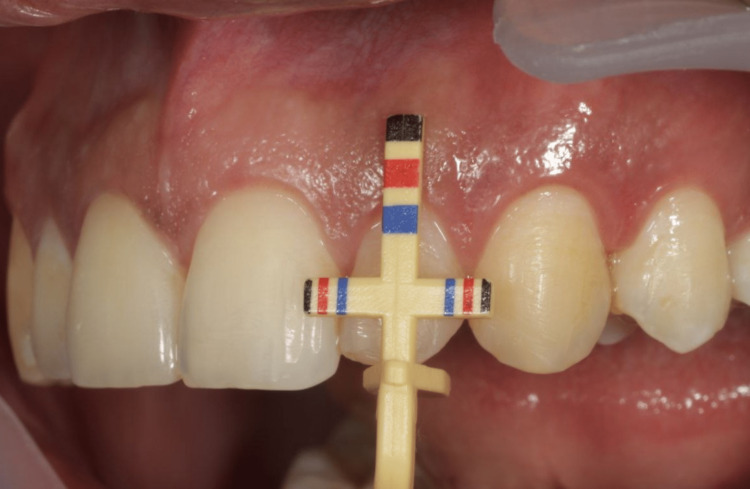
The variation between the color coding on the Chu’s gauge and the dimension of lateral incisors

**Figure 4:**
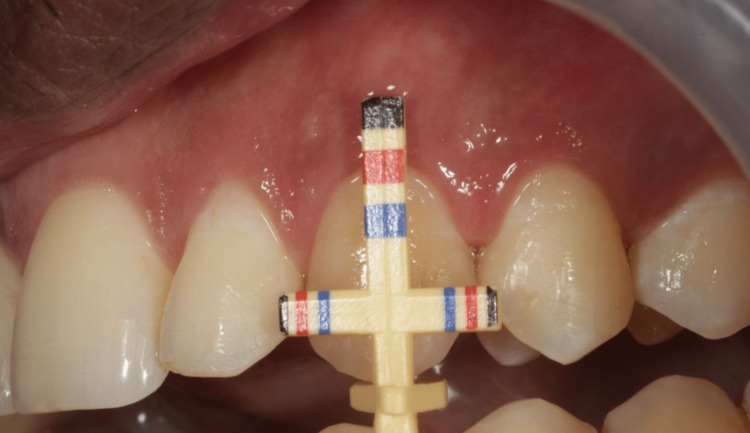
The variation between the color coding on the Chu’s gauge and the dimension of the canine

Maxillary anterior teeth - central incisors (Figure 2), lateral incisors (Figure 3), and the canine (Figure 4) - on both the right and left side were evaluated using the T-bar tip of Chu’s proportion gauge to assess the height and width. The subjects whose teeth width and height proportion coincided with the Chu’s proportion gauge were considered as coinciding and those who did not were considered as not coinciding.

**Figure 5:**
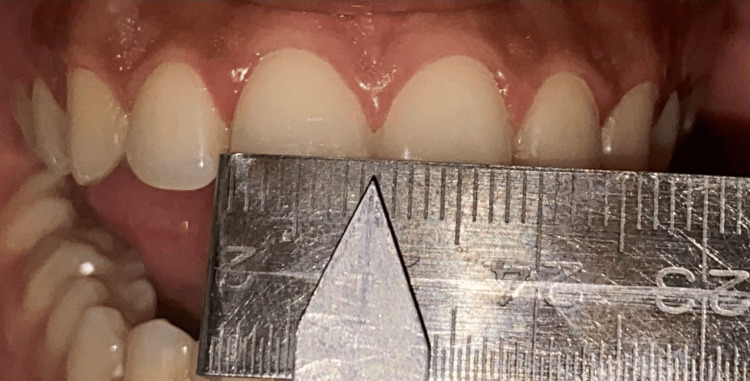
The measurement of central incisor width by using the custom-made caliper

To assess the height and width, a custom-made caliper was used, which was made by using a graduated metallic ruler to which a ledge was added to enhance the stability over the incisal edge of the teeth for accurate measurement. The custom-made caliper (Figure 5) was used to measure the height and width of anterior teeth on both the left and right sides in millimeters. The values obtained were tabulated. The custom-made caliper and the Chu’s gauge were disinfected and autoclaved. The values obtained were subjected to statistical analysis.

## Results

Of the 362 subjects included in the study, 141 (39%) had the central incisor dimension coinciding with the color-coding red band on the left side, and 146 (40.3%) subjects had it coinciding on the right side. In around 60% of the population, the dimension of the central incisor did not coincide with the color coding on Chu’s gauge. In 39 (10.8%) subjects, the lateral incisor dimension coincided with the color-coding blue band on the left side, and in 35 (9.7%) subjects, it coincided on the right side. In 23 (6.4%) subjects, the canine dimension coincided with the color-coding yellow band on the left side, and in 19 (5.2%) subjects, it coincided on the right side (Table 1).

**Table 1:** The proportion of values coinciding and not coinciding with the values on Chu’s gauge (N=362)

	Chu's gauge, left, n (%)			Chu's gauge, right, n (%)		
	Central	Lateral	Canine	Central	Lateral	Canine
Not coinciding	221 (61.0%)	323 (89.2%)	338 (93.4%)	216 (59.7%)	326 (90.1%)	343 (94.8%)
Coinciding	141 (39%)	39 (10.8%)	23 (6.4%)	146 (40.3%)	35 (9.7%)	19 (5.2%)

The average height of the left maxillary central incisors was around 10.24 mm while the width was around 8.94 mm. The average height of the right maxillary central incisors was around 10.14 mm and the width was around 8.94 mm. The average height of the left lateral incisors was around 8.6 mm and the width was around 7.51 mm. The average height of the right lateral incisors was 8.59 mm and the width was 7.62 mm. The average height of the left canine was around 9.04 mm and the width was around 8.14 mm. The average height of the right canine was 8.59 mm and the width was 8.99 mm width (Table 2).

**Table 2:** The average height and width of both the right and left sides of the maxillary anterior teeth (N=362) SD: standard deviation

Height, left, mm, mean (SD)			Height, right, mm, mean (SD)			Width, left, mm, mean (SD)			Width, right, mm, mean (SD)		
Central	Lateral	Canine	Central	Lateral	Canine	Central	Lateral	Canine	Central	Lateral	Canine
10.24 (1.073)	8.62 (1.085)	9.04 (1.173)	10.14 (2.223)	8.59 (1.025)	8.998 (1.156)	8.94 (0.759)	7.51 (0.839)	8.14 (0.725)	8.94 (0.654)	7.62 (1.017)	8.33 (0.764)

## Discussion

A smile is a curve that sets everything straight. Many dental and facial traits differ based on individuals' geographical regions and historical backgrounds. Being well-versed in data on teeth-related norms in various populations can be valuable to dentists while restoring teeth [10]. The current studies on demographic data focus on evaluating different population groups because the golden percentage and golden proportion are no longer valid in all ethnic groups.

The most dominant teeth related to the smile curve are the maxillary anteriors; determining the right tooth size leads to improved esthetics as well as treatment results [10]. Dental treatment is influenced by various micro and macro elements, which are inseparable and affect one another. The macro feature highlights include tooth size, shape, form, and proportion while tooth shade, color, surface texture, and translucency come under micro features. The dental specialist should consider the patient's subjective needs while planning a smile design along with the objectives to be achieved [15,16,17].

The studies by Shetty and Mootha et al. [2] evaluated the presence of RED proportion in various populations and reported that RED proportion was not seen in natural dentition. Alqahtani et al. (2021) [3] conducted a study to measure and analyze the dimensions of the maxillary anterior teeth and their relative width proportions in a Saudi subpopulation and it involved a population of 180 patients with intact maxillary anterior teeth. The study concluded that the mean width for the central incisor was 8.74 mm (men: 8.89 mm, women: 8.60 mm), that for the lateral incisor was 6.64 mm (men: 6.79 mm, women: 6.49 mm), and canine was 7.82 mm (men: 8.01 mm, women: 7.63 mm). The recorded mean lengths for these teeth were 9.84 mm (men: 10.04 mm, women: 9.64 mm), 8.09 mm (men: 8.30 mm, women: 7.89 mm), and 9.08 mm (men: 9.48 mm, women: 8.69 mm).

The ethnic characteristics specific to populations are associated with variations in the reported studies. In this study, the gingival zenith was considered as the landmark to check the height of the tooth. The height was measured from the gingival zenith to the incisal edge using the T-bar of Chu’s esthetic gauge and custom-made caliper. Though CEJ is the most accurate landmark, due to clinical constraints, the gingival zenith was used as the landmark. The width was measured from the mesial contour of the tooth to the distal contour by using a custom-made caliper [9,11,18].

The results of the current study showed that the average height and width of the left central incisors were 10.24 and 8.94 mm respectively, whereas the height and width of the right central incisor were 10.14 and 8.14 mm.

Limitations

This study has a few limitations. It was conducted in a specific demographic area of southwestern India. Hence, the results may not be generalizable to other geographical areas.

## Conclusions

The evaluation of dental dimensions and their correspondence with color coding on Chu's gauge provided valuable insights into the variability and distribution of tooth dimensions in the study population. Within the limitations of the study, it can be concluded that the average markings present on Chu's gauge did not coincide with the height and width of the studied population. It can be said that the dimension of anterior teeth is specific to the demographic area and hence the average dimension for that specific area has to be determined. Further research in this field could focus on exploring the factors contributing to the observed variations in teeth dimensions, such as genetic influences, ethnicity, and environmental factors. Comprehending the underlying determinants of tooth size and shape variations can contribute to improved dental treatment planning and the development of more accurate dental prosthetics and restorative techniques tailored to individual patients.
